# The relationship between quality of life and some mental problems in women with gestational diabetes mellitus (GDM): a cross-sectional study

**DOI:** 10.1186/s12888-024-05960-4

**Published:** 2024-07-18

**Authors:** Soheila Nazarpour, Masoumeh Simbar, Zahra Kiani, Neda Khalaji, Mobina Khorrami Khargh, Zahra Naeiji

**Affiliations:** 1 Department of Midwifery, Chalous Branch, Islamic Azad University, Chalous, Iran; 2 Midwifery and Reproductive Health Research Center, Shahid Beheshti University of Medical Sciences, Tehran, Iran; 3 Department of Midwifery and Reproductive Health, School of Nursing and Midwifery, Shahid Beheshti University of Medical Sciences, Tehran, Iran; 4 Department of Obstetrics and Gynecology, School of Medicine, Mahdieh Hospital, Shahid Beheshti University of Medical Sciences, Tehran, Iran

**Keywords:** Quality of life, Gestational diabetes mellitus, Mental health, Depression, Anxiety, Stress

## Abstract

**Background:**

Women with medical problems during pregnancy, including women with Gestational Diabetes Mellitus (GDM), experience an increased prevalence of mental health disorders which can affect their quality of life. This study aimed to assess the relationship between GDM-related quality of life and depression, anxiety, and stress.

**Methods:**

This analytical cross-sectional study was performed on 150 women with GDM. The participants were selected using a multi-stage sampling including quota and then randomized method from maternal care centers affiliated with Shahid Beheshti University of Medical Sciences, Tehran-Iran. The data were collected using a personal information questionnaire, the GDM-related quality of life questionnaire (GDMQoL-36), and the depression, anxiety, and stress scale (DASS). The data were analyzed using SPSS-23 software and statistical tests of coefficient Spearman’s correlation, *t*-test, analysis of variance, and multiple linear regression.

**Results:**

The mean ± SD score for the GDM-related quality of life and the DASS scale were 55.51 ± 8.87 and 27.12 ± 19.43%, respectively. Different degrees of depression, anxiety, and stress were present in 40, 61.3, and 42% of women, respectively. The total score of GDM-related quality of life had a significant negative correlation with the total score of DASS and the scores of the subscales including depression, anxiety, and stress (*P* < 0.001). There were significant correlations between the total score of GDM-related quality of life with age, BMI, length of marriage, educational level of the woman and her spouse, the occupation of the woman and her spouse, income, and economic class of the family. Multiple linear regression revealed that depression, education, and job are predictive factors for GDM-related quality of life.

**Conclusion:**

GDM-related quality of life is related to some mental disorders. Therefore, it is important to consider the mental health promotion of pregnant women with GDM in future prenatal health programs to improve their quality of life. This also shows the importance of integrating mental health promotion strategies to enhance the quality of life of pregnant women with GDM.

## Introduction

Gestational diabetes Mellitus (GDM) is the most common medical complication of pregnancy [1]. According to the 2024 American Diabetes Association (ADA), Gestational diabetes mellitus is defined as diabetes diagnosed in the second or third trimester of pregnancy that was not overt diabetes before gestation or other types of diabetes occurring throughout pregnancy, such as type 1 diabetes. According to the ADA report, type 1 diabetes is caused by autoimmune beta-cell destruction and usually leads to absolute insulin deficiency in adults, and type 2 diabetes is caused by a non-autoimmune progressive loss of adequate β-cell insulin secretion, frequently on the background of insulin resistance and metabolic syndrome [2].

This disease is usually diagnosed in weeks 24 to 28 [3]. The global prevalence of GDM is estimated at 10.13% and evidence suggests that diabetes in all forms, especially gestational diabetes, is increasing as one of the main metabolic disorders in pregnancy [4, 5]. So, GDM is known as one of the fastest-growing forms of diabetes due to the increase in obesity rates and maternal age worldwide [6]. The latest documents from the International Diabetes Federation show that in 2021, 16.7% (1 in 6) of live births worldwide were affected by maternal hyperglycemia during pregnancy, 80.3% of which were due to GDM [7–9].

This disease affects approximately 6% of pregnancies in Iran, with an estimated prevalence of 1.3 to 18.6% [10]. In a meta-analysis in Iran, the overall prevalence of GDM in 2015 was estimated at 3.4% [11].

GDM is associated with adverse maternal outcomes such as increased risk of cesarean section, preeclampsia, develop type 2 diabetes, cardiovascular disease, malignant tumors, ophthalmic diseases.

renal disease, dyslipidemia and postpartum metabolic disorders; and fetal outcomes including increasing the risk of LGA and macrosomia, shoulder dystocia, preterm labor, stillbirth, infant mortality, neonatal complications, fetal hyperglycemia, hyperbilirubinemia, neonatal respiratory distress syndrome, impaired neurodevelopment, increased risk of type 2 diabetes, obesity, cardiovascular disease, and mental disorders [1, 12–21]. Maternal blood glucose transfers to the fetus through the placental circulation and causes fetal hyperglycemia [20], which may lead to LGA and macrosomia. Fetal hyperglycemia causes fetal tissue disproportion, including increased fetal fat tissue, thickening of the skin folds, and increased shoulder-to-head ratio. Therefore, these infants are at risk for dystocia and shoulder fractures due to the anthropometric alterations [15]. Besides, increased rates of preterm labor and cesarean section are from other important complications of GDM, which can lead to stillbirth and infant mortality [16].

Babies born neonates of women with GDM are at high risk of hypoglycemia, hyperbilirubinemia, and neonatal respiratory distress syndrome, as well as prolonged length of stay in the neonatal intensive care unit [19]. In addition, these infants are also at risk for poor long-term health outcomes, including impaired neurodevelopment, difficulty maintaining a normal body mass index (BMI), and increased risk of type 2 diabetes, obesity, cardiovascular disease, and mental disorders [17]. Compared to women with normal pregnancies, pregnant women with GDM are more likely to develop type 2 diabetes [21], cardiovascular disease, malignant tumors, kidney disease and ophthalmic diseases, dyslipidemia, and postpartum metabolic disorders [12–14, 17, 18].

This clinical condition potentially has a negative effect not only on medical outcomes but like other chronic diseases, it can negatively affect almost all aspects of the patient’s life. It often leads to the deterioration of the patient’s physical and mental health, changes in lifestyle and adaptation to the disease, as well as changes in physical, professional, and social activities, as well as values. All this also affects the patient’s quality of life [22–25].

The risks and adverse consequences mentioned above force pregnant women with GDM not only to bear the physical and mental discomfort of the disease but also to worry about the safety and prognosis of the fetus. In addition, the behavioral restrictions caused by the disease have effects on the social activities and work life of these pregnant women, and the costs of treating the disease increase the economic burden of their families to different degrees [26]. All these results seriously affect the quality of life of pregnant women with GDM [17–19, 26–29].

Mental disorders of pregnant women, especially the psychological condition of women with GDM, as a high-risk group, attracted much attention from researchers around the world. Studies conducted in this population indicate that apart from physiological factors, anxiety and depression are also associated with GDM [27]. Evidence suggests that there may be a bidirectional relationship between gestational diabetes and anxiety and depression [27]. Anxiety and depression can lead to chronic hypothalamic-pituitary-adrenal hyperactivity, which leads to increased cortisol secretion and insulin resistance [30] and increased risk of GDM in pregnant women. On the other hand, these patients face many obstacles and challenges, such as mental stress, fear of disease, and worries during pregnancy, and they feel more anxious about the possibility of developing diabetes and their neonatal health (the effect of insulin or diet on the fetus) [27, 31]. A possible physiologic mechanism for this significant association could be linked to the secretion of cortisol and expression of certain inflammation markers in pregnancy, which are in turn associated with hyperglycemia and insulin resistance [32]. At the same time, a diagnosis of GDM may increase the risk of antepartum or postpartum depression through a reverse mechanism [33]. Several studies have shown that women with medical problems during pregnancy, including women with GDM, report higher levels of symptoms of depression, anxiety, and stress compared to women without complications [27, 34–39]. In a study on 526 women with GDM in Malaysia, it was found that among women with GDM, the prevalence of anxiety symptoms was the highest (39.9%), followed by depressive symptoms (12.5%) and stress symptoms (10.6%) [40]. Also, Hinkle et al.‘s study showed that the probability of depression in women with GDM is 2 to 4 times higher than pregnant women without GDM [34].

Quality of life (QoL) is the most important concept studied in health care research. With the increase in life expectancy and the prevalence of chronic diseases, there is a need to pay attention to the quality of life. Assessment of quality of life helps to improve the health status of patients and the quality of care provision [41]. Quality of life therapy empowers people to actualize their knowledge, attitudes and values [42].

In recent years, healthcare and clinical researchers have concentrated on the concept of quality of life to assess the healthcare challenges in chronic diseases [43, 44]. Quality of Life is defined as an individual’s perception of their position in life in the context of the culture and value systems in which they live and about their goals, expectations, standards, and concerns [45]. GDM also has physical, social, mental, and psychological consequences that can affect the quality of life of women [46]. Thus, increasing the quality of life through reasonable interventions is considered as important as metabolic control and prevention of complications in GDM care and treatment procedures. Therefore, quality of life assessment should be implemented as a clinical standard in GDM care [44].

The quality of life of women with GDM is affected by several factors [29]. Today, there is increasing attention to the quality of life, and researchers investigated many factors to identify the effective factors. According to studies, several factors affect the quality of life of women with GDM. Individual-specific variables including demographic variables such as age [43], level of education and BMI [47], variables related to pregnancy and disease, social factors, and psychological factors are important factors that affect the quality of life of pregnant women with GDM [29, 43]. Undoubtedly, identifying the factors affecting the quality of life in diabetic patients improves the health of patients and increases their survival [43]. An important group of these factors are mental disorders because GDM can have negative effects on maternal mental health and thereby affect the quality of life [25]. Therefore, this study aimed to assess the relationship between depression, anxiety, and stress with the quality of life of women with GDM.

## Methods

### Study design

This was a correlational cross-sectional study.

### The participants

The samples were 150 pregnant women affected by GDM and referred to the prenatal care clinics of the hospitals affiliated with Shahid Beheshti University of Medical Sciences (SBMU), Tehran-Iran.

Inclusion criteria included diagnosis of with at least one abnormal value ≥ 92, 180, and 153 mg/dl for fasting, one-hour, and two-hour plasma glucose concentration respectively, after a 75 g oral glucose tolerance test in 24–28 weeks of pregnancy [48, 49]. The exclusion criteria were the incomplete responses to the questionnaires. However, there was no missing in sampling because the information was collected by Google form, and answering the questions was mandatory, so the questionnaires could not be submitted without responding to all questions.

### Sampling

A multi-stage sampling including quota and then randomized method was used to recruit the subjects of the study. Firstly, four hospitals in the north, south, east, and west of Tehran affiliated with SBMU were selected. Then, quota sampling was used to recruit samples from the prenatal care clinics of the selected hospitals including Taleghani, Mahdieh, Emam-hossein, and Shohada. Hospitals. Following the total sample size calculation, the number of participants was distributed based on the monthly average number of clients with GDM who were visited in each clinic. At that time, the samples were randomized using the Excel random selection option from the women with the eligibility criteria. Then they were informed about the objectives of the study and signed the electronic informed consent form before completing the questionnaires, and, finally, the questionnaires were completed electronically by the participants.

The number of samples for the study was calculated at 146 using the following formula. The total sample size N = [(Zα + Zβ)/C]2 + 3 = 146, considering α (two-tailed) = 0.05, β = 0. 20 and *r* = 0.23 (stress and quality of life) [25, 43] and therefore considering the standard normal deviate for α = Zα = 1.96, the standard normal deviate for β = Zβ = 0.84 and C = 0.5 * ln[(1 + r)/(1-r)] = 0.23.

### Tools for data collection

The tools for data collection were 3 questionnaires including a personal information questionnaire, a valid and reliable questionnaire to assess Quality of life in Gestational Diabetes Miletus (GDMQoL-36) designed by Mokhleshi et al. [50], and the Depression, anxiety, and stress scale (DASS) questionnaire [51].

#### The personal information questionnaire

The questionnaire Contains items related to socio-demographic and fertility information. It included 21 questions about the participant’s age, education, income, employment status weight, height, duration of the marriage, gravida, parity, abortion, unwanted or unwanted pregnancy, desired sex of the fetus, gestational age, as well as the history of gestational diabetes, history of preterm labor and history of stillbirth, and also about the GDM and the treatment protocols. All the questionnaires were prepared as the Google form and were electronically filled up after giving informed consent.

#### Gestational diabetes miletus-related quality of life questionnaire (GDMQoL-36)

GDMQoL-36 is developed to assess the quality of life of women with GDM. It consists of 36 questions in 5 domains (concerns about high-risk pregnancy, perceived constraints, complications of GDM, medication and treatment, and support).

The items in the domains of concerns about high-risk pregnancy, perceived constraints, complications of GDM, and medication and treatment are scored by a 5-point Likert scale 1 to 5 strongly agree to strongly disagree). There was an exception for item 30, “I adjust insulin dose based on my blood glucose” which is scored 5 to 1 for strongly agree to strongly disagree. In the domain of support, the answers are scored 5 to 1 for strongly agree to strongly disagree. For the participants who do not receive Insulin, the scores of these questions are considered 3 (Neutral).

The total score of the instrument is computed by calculating the average of the total modified scores of the instrument. The total score of the questionnaire, based on the above explanations is 36–180, with higher scores representing higher quality of life. Because of the diversity of the domains and the scales, a standard 0 to 100 method of scoring was used for better understanding and comparison of the scores of the domains. To convert the scores from 0 to 100, the following formula was used. Adjusted score = (raw score-minimum /maximum -Minimum) *100.

GDMQoL-36 is a standard questionnaire with S-CVI and S-CVR 0.99 and 0.73, respectively. Factor analysis using varimax rotation indicated that the 5 factors can explain 46.68% of the variance. Also, a significant convergent validity was demonstrated between GDMQoL-36 and the “Diabetes Clients Quality of Life questionnaire” (DCQOL) (*r* = 0.64) [52]. The internal consistency of the GDMQoL-36 was shown by Cronbach’s alpha 0.93 and its test-retest stability was demonstrated by an intra-class correlation coefficient of 0.95 [50]. This questionnaire is developed and psychometrically assessed in Iran.

#### Depression, anxiety, and stress scale questionnaire (DASS)

The Depression Anxiety Stress Scale 21 (DASS-21) is a short form of Lovibond and Lovibond’s (1995) 42-item measure of depression, anxiety, and stress (DASS) [51]. The shortened 21-item scale performs as well as the 41-item scale and is considered the preferred version of the scale [53, 54]. The DASS-21 questionnaire includes 3 subscales and contains 21 questions and evaluates depression, anxiety, and stress with 7 questions for each subscale, and scoring based on a 4-point scale from 0 to 3. The final score of each subscale is obtained through the sum of the scores of the related questions. The items 1, 6, 8, 11, 12, 14, and 18 measure stress, and the items 3, 5, 10, 13, 16, 17, and 21 assess depression, and also the items 2, 4, 7, 9, 15, 19, and 20 measure anxiety. The scores range from 0 to 21 for each subscale, and the total score ranges from 0 to 63. A higher score indicates more depression, anxiety, and stress. The scoring method is that each question is considered from 0 (does not apply to me at all) to 3 (completely applies to me). Since DASS-21 is the shortened form of the main scale (42 questions), the final score, for each subscale should be doubled (the total score is between 0 and 126 and the score of each subscale is between 0 and 42) [51, 55, 56]. Then, the responders are classified into normal, mild, moderate, severe, and very severe groups, based on the scoring results and according to Table 1 [57].

**Table 1:** DASS severity ratings (multiply summed scores by 2)

Severity	Depression	Anxiety	Stress
Normal	0–9	0–7	0–14
Mild	10–13	8–9	15–18
Moderate	14–20	10–14	19–25
Severe	21–27	15–19	26–33
Extremely Severe	28+	20+	33+

The validity of the short form of DASS-21 was evaluated by Crawford and Henry. The reliability of the DASS-21 was confirmed by Cronbach’s alpha at 0.88 for depression, 0.82 for anxiety, 0.90 for stress, and 0.93 for the total scale [54].

The Persian version of the questionnaire was validated by Sahibi et al. and the internal consistency of the test was determined to be satisfactory and was almost equal to the internal consistency of the original version of the DASS created by Lavibond and Lavibond in 1995. In the study of Sahebi et al., the internal consistency of the DASS scales was calculated using Cronbach’s alpha at 0.77 for the depression subscale, 0.79 for the anxiety subscale, and 0.78 for the stress subscales [58]. The Persian version was used in this research.

### Statistical analysis

The data was extracted from the Google form in the Excel software that was converted to SPSS-23. The normality of the variables was examined using the Kolmogorov-Smirnov test. Then, the data were analyzed using t-test, ANOVA, Spearman correlation tests, and linear multiple regression analysis. Multiple linear regression was performed by a backward stepwise method. *P* values less than 0.05 were considered significant.

## Results

One-hundred fifty women with GDM with an average age of 31.44 ± 6.64 (Mean ± SD) years and gestational age of 30.77 ± 6.09 weeks participated in this study. Among these women, 45.3% had a history of GDM in a previous pregnancy, and 50% of them used insulin. The sociodemographic characteristics of women are presented in Table 2.

**Table 2:** The demographic and reproductive characteristics of the women with gestational diabetes mellitus (GDM) (*n* = 150)

Variables		Mean ± SD/ *N* (%)
Age (years)		31.44 ± 6.64
BMI (Kg/m^2^)		33.36 ± 31.03
Duration of marriage (years)		8.76 ± 6.71
Education	Under diploma	101 (67.3)
	Diploma	30 (20.0)
	Academic	19 (12.7)
Spouse’s Education of	Under diploma	95 (63.3)
	Diploma	31 (20.7)
	Academic	24 (16.0)
Occupation	Housewife	99 (66.0)
	Employed	51 (34.0)
Spouse’s Occupation	Worker	40 (26.7)
	Employee	32 (21.3)
	Freelance job	68 (45.3)
	Retired or unemployed	10 (6.7)
Family Economic Status	Low	75 (50.0)
	Moderate	56 (37.3)
	High	19 (12.7)
Family income	Inadequate	65 (43.3)
	Adequate	85 (56.7)
Insulin intake	Not taking insulin	75 (50.0)
	Once	20 (13.3)
	Twice	31 (20.7)
	Three times and more	24 (16.0)
**Reproductive History**		Mean ± SD/ N (%)
Gravida	0	1 (0.7)
	1–2	96 (63.3)
	3–4	41 (27.3)
	≥ 5	13 (8.7)
Abortion	0	108 (72.0)
	1–2	35 (23.3)
	≥ 3	7 (4.7)
Gestational Age	First trimester	2 (1.3)
	Second trimester	52 (34.7)
	Third trimester	96 (64.0)
History of preterm labor		21 (14.0)
History of GDM		68 (45.3)
History of stillbirth		13 (8.7)
Current unwanted pregnancy		57 (38.0)

The total score for GDMQoL-36 was 55.51 ± 8.87% (Mean ± SD). The highest score was related to the domain of support (72.53 ± 16.51%) and the lowest score belonged to Perceived constraints (39.06 ± 20.76%) (Table 3).

**Table 3:** The mean and standard deviation of different domains of women’s quality of life related to gestational diabetes Miletus

Variables	Domains	Score (36–180) (Mean ± SD)	Score (0-100) (Mean ± SD)
GDMQ-36	Concerns about high-risk pregnancy	42.57 ± 8.47	71.76 ± 19.26
	Perceived constraints	**20.50 ± 6.64**	39.06 ± 20.76
	Complications of GDM	16.62 ± 5.28	44.25 ± 22.01
	Medication and treatment	12.84 ± 2.85	39.20 ± 14.26
	Support	**23.41 ± 3.96**	72.53 ± 16.51
	Total GDM	115.94 ± 12.77	55.51 ± 8.87

The total score for the DASS-21 scale was 27.12 ± 19.43%. The scores for the depression, anxiety, and stress subscales were 22.51 ± 22.05, 23.17 ± 16.93, and 35.68 ± 23.82%, respectively. Findings revealed that the participants are experiencing different degrees of depression (40%), anxiety (61.3%), and stress (42%) (Table 4).

**Table 4:** Frequency depression, anxiety and stress scale 21 (DASS-21) in gestational diabetes Miletus

DASS-21 Scoring (Grades)	Depression *n* (%)	Anxiety *n* (%)	Stress *n* (%)
Normal	90 (60.0)	58 (38.7)	87 (58.0)
Mild	13 (8.7)	20 (13.3)	23 (15.3)
Moderate	23 (15.3)	36 (24.0)	18 (12.0)
Severe	15 (10.0)	17 (11.3)	10 (6.7)
Extremely Severe	9 (6.0)	19 (12.2)	12 (8.0)
**Score (0–21)(Mean ± SD)**	**9.45 ± 9.26**	**9.73 ± 7.11**	14.99 ± 10.00
**Score (0-100) (Mean ± SD)**	**22.51 ± 22.05**	**23.17 ± 16.93**	35.68 ± 23.82
Total Score (0–63) (Mean ± SD): 34.17 ± 24.48			
Total Score (0-100) (Mean ± SD): 27.12 ± 19.43			

The correlations between the total score of GDMQoL-36, with the total score of DASS-21, and its subscales are shown in Table 5. As the table shows, there were significant negative correlations between the total score of GDMQoL-36, with the total score of DASS-21, and depression, anxiety, and stress subscales (*P* < 0.001).,

**Table 5:** Correlations between women’s quality of life-related to gestational diabetes Miletus (GDMQoL-36) with depression, anxiety, and stress (DASS-21) (*n* = 150)

GDMQoL-36						
Variables	Concerns about high-risk pregnancy	Perceived constraints	Complications of GDM	Medication and treatment	Support	Total GDMQ
	r	r	r	r	r	r
Depression	-0.061	-0.322^***^	-0.461^***^	-0.347^***^	-0.076	-0.469^***^
Anxiety	0.017	-0.215^**^	-0.550^***^	-0.282^***^	0.014	-0.350^***^
Stress	0.003	-0.181^*^	-0.370^***^	-0.305^***^	-0.090	-0.301^***^
Total (DASS)	-0.008	-0.253^**^	-0.478^***^	-0.338^***^	-0.059	-0.393^***^

^*^P < 0.05,^**^P < 0.01, ^***^P < 0.001; Test: Spearman correlation coefficients; GDMQoL-36: Quality of Life in Gestational Diabetes Miletus

Also, the score of Perceived constraints, Complications of GDM, and Medication and treatment dimensions of quality of life had a significant negative correlation with the total score of DASS-21 and the scores of depression, anxiety, and stress.

The relationships between GDMQoL-36 and the sociodemographic characteristics are shown in Table 6. The results showed significant negative correlations between age (*P* = 0.016), BMI (*P* = 0.005), and length of marriage (*P* = 0.045) with the total score of GDMQoL.

**Table 6:** The correlation between personal factors and GDMQoL-36 (*n* = 150)

Personal factors	Quality of life (total score of GDMQoL-36)			
	Test	***p***	95% Confidence Interval	
			Lower	Upper
Age (years)	*r*^*s*^= -0.197	0.016	-0.344	-0.044
BMI (Kg/m^2^)	*r*^*s*^= -0.229	0.005	-0.386	-0.080
Duration of marriage (years)	*r*^*s*^= -0.164	0.045	-0.320	-0.005
Gravida	*r*^*s*^= -0.149	0.070	-0.311	0.026
Abortion	*r*^*s*^= -0.034	0.678	-0.188	0.124
Gestational Age [97] ^S^	*r*^*s*^=-0.015	0.852	-0.177	0.140
Education	ANOVA*	< 0.001		
Education of Husband	ANOVA*	< 0.001		
Occupation	*t-Test*	0.007		
Husband Occupation	ANOVA*	0.003		
Family income	*t-Test*	0.002		
Family Economic class Family	ANOVA*	0.001		
History of GDM	*t-Test*	0.062		
Insulin intake**	ANOVA*	0.168		
History of preterm labor	*t-Test*	0.247		
History of stillbirth	*t-Test*	0.237		
Current unwanted pregnancy	*t-Test*	0.382		

S: Spearmen Correlation Test

* ANOVA was performed to compare means between GDMQoL of several groups, and the CI in the significant cases was presented

** GDMQoL was not significant between different Insulin consumption users and was not significantly different between the groups

Also, the ANOVA test revealed a significant relationship between GDMQoL, with the women’s education and their spouse’s education (*P* < 0.001). Also, Tamhane’s T2 post hoc test showed that the score GDMQoL is higher among participants with higher education (*P* < 0.05).

Independent *t*-tests showed that working women had a higher GDMQoL score than housewives (*P* = 0.007). Also, the ANOVA test disclosed a significant difference in the scores of GDMQoL based on the husband’s occupation (*P* = 0.007) and Scheffe’s post hoc test revealed that the GDMQoL score was higher in women whose husbands were employees than in the women whose husbands were workers (*P* < 0.05).

Performing the *t*-test showed that women with GDM with sufficient family income had a higher GDMQoL score than women with insufficient family income (*P* = 0.001). Also, the ANOVA test showed a significant difference in GDMQoL based on the economic class of the family (*P* = 0.001) and Scheffe’s post hoc test revealed more GDMQoL scores in women with high economic class compared to the two groups with Middle and low economic class (*P* < 0.05). The rest of the sociodemographic variables did not show a significant relationship with the GDMQoL.

The assumption for the multiple linear regression model was that GDMQoL-36 was related to depression, anxiety, and stress. In multiple linear regression, the GDMQoL-36 score was considered the dependent variable, and scores of DASS were the main variables whose relation to the GDMQoL-36 score was measured. Age, BMI, duration of marriage, education (women and husband), occupation (women and husband), income, and economic class were included in regression models by stepwise method, as they were considered potential confounding variables. In our regression analyses, the R^2^ = 0.372, which showed that 37.2% of the outcome variable (score of GDMQoL-36) was explained by the variables included in the regression model. The interactions between confounding variables were assessed. However, these interaction terms were not included in the final model as they were not statistically significant.

The results of multiple linear regression based on the Stepwise method showed that depression, education, and occupation are predictive factors for the total GDMQoL score. So for each unit increase in depression score, the total GDMQoL score decreases by 0.689 units (*P* < 0.001). Also, the total GDMQoL score in working women is 4.233 higher than that of housewives (*P* = 0.022). Also, with an increase in education level, the total GDMQoL score increases by 4.872 (*P* < 0.001) (Table 7).

**Table 7:** Predictors of quality of life (total score of GDMQ-36)

Predictors	Multiple linear regression					
	B	Beta	t	*p*-value	95.0% Confidence Interval for B	
					Lower	Upper
Depression	-0.689	-0.499	-7.614	< 0.001	-0.868	-0.510
Education	4.872	0.271	3.994	< 0.001	2.461	7.282
Occupation	4.233	0.158	2.322	0.022	0.631	7.836
*R* = 0.610	**R Square = 0.372**		**Adjusted R Square = 0.360**			

^a^Classification of education: 1. Under diploma, 2:Diploma, 3:Academic

^b^Classification of occupation:1. Housewife, 2. Employed

## Discussion

This study showed there is a strong negative correlation between GDM-related quality of life (GDMQoL) with mental disorders including depression, anxiety, and stress. Among mental disorders, depression is a predictor of the GDMQoL. Stress, depression, and anxiety are the most important psychological reactions of an individual who is diagnosed with a new disease such as gestational diabetes [59]. Some studies also showed the significant role of intervening psychosocial factors, such as depression and stress in the quality of life of diabetic patients [43, 60]. QOL of women with GDM had been severely affected by concerns about a high-risk pregnancy [46].

The finding demonstrated a strong significant negative correlation between GDMQoL and depression, and depression was a predictor for the total score for GDMQoL, so for each unit increase in depression score, the total GDMQoL score decreased by 0.689 units. This result is inconsistent with other studies [27, 29, 61–64]. Depression is a common complication in the perinatal period which is associated with an increased risk of adverse pregnancy outcomes [65, 66]. In a systematic review, OuYang et al. showed that depression is a risk factor for poor quality of life in pregnant women with GDM [27]. Depression not only leads to hormonal imbalance and increased blood sugar in pregnant women but also increases the incidence of cesarean section and adverse maternal-fetal/neonatal consequences [27]. The higher prevalence of depressive symptoms in women with GDM may be related to a less healthy lifestyle in these women [62]. The consequences of prenatal depression are not limited to pregnancy and childbirth itself, but may also have postnatal significant negative outcomes [62]. Depressed women with GDM decrease the use of social support and they have serious concerns about the disease and treatment, which in turn increases the development of depression, and forms a vicious circle of further decreasing quality of life [63]. In a study on 1843 Belgian women, Minschart and colleagues found that women with prenatal depressive symptoms are more likely to develop GDM, and these women often remain depressed during the postpartum period and have a lower quality of life [62].

The present study showed a strong significant negative correlation between GDMQoL and anxiety. This result is consistent with some studies [61, 63, 67]. Mokhlesi and colleagues showed that worry during pregnancy was significantly higher in women with GDM compared to low-risk pregnant women. Among the different dimensions of GDMQoL, concerns about high-risk pregnancy, such as concerns about childbirth and the neonate, are the most critical issues in gestational diabetes [31] and the strongest predictor of their quality of life [50]. Women reported concerns about their fetus and neonate health, preterm labor and reduced fetal movements, intrauterine growth restriction, and stillbirth due to GDM [31]. These concerns cause anxiety that affects mental health and the quality of life [39]. In a study on 526 pregnant women with GDM in Malaysia, Lee et al. indicated that women with a family history of depression or anxiety compared to those who did not have this history were more likely to suffer from poor to moderate quality of life [61].

The results showed a strong significant negative correlation between the scores for GDMQoL and stress. The relationship between stress and low quality of life is demonstrated in the study of Long and colleagues on 465 Chinese women with a history of GDM [35]. Indeed, pregnancy is a stressful condition for women which can be exacerbated by high-risk pregnancy [31, 68]. GDM diagnosis is usually unexpected and may increase negative experiences and perceptions during pregnancy [59]. Furthermore, the quality of life of women with GDM may be affected by concerns about maternal and fetal/child health, as well as by the feeling of losing control over their health [69, 70]. Frequent metabolic changes caused by GDM require regular visits to doctors and medical treatment, which can cause stress in sensitive situations such as pregnancy [71], and also can hurt the quality of life.

The average score of GDMQol was 55.51 ± 8.87%, and the highest and the lowest scores were related to “Support” and “Perceived constraints”, respectively. The results of the present study are similar to other studies that show that the average quality of life of pregnant women decreases after the diagnosis of GDM [29, 46, 61, 72]. The quality of life of pregnant women with GDM is usually low, and about a quarter of pregnant women have a poor quality of life [29]. In the study of Simbar and colleagues, which was conducted on 200 women with GDM, the quality-of-life score was 46.83, and the highest and lowest scores belonged to the subscales of “support” and “concern about high-risk pregnancy”, respectively [46]. This low quality of life may be due to possible serious risks and adverse consequences for women with GDM and their babies. So women with GDM not only have to bear the physical, psychological, economic, and social problems of this disease but also, they are worried about the child’s health which seriously affects their quality of life [29].

The present study showed a significant negative relationship between GDMQoL and women’s age. This result is consistent with the results of some other studies showing significant negative effects of age on quality of life in diabetic patients [43, 67, 72–74]. In a population-based cross-sectional study of 13,358 pregnant women in China, Liu et al. showed that GDM and advanced maternal age were associated with decreased general health as one of the domains of quality of life [67]. As age increases, the adverse outcomes of pregnancy increase, which can negatively affect the quality of life [43].

There was also a significant negative relationship between BMI and GDMQoL. This relationship was found in other studies [47, 75–77]. In a Path analysis by Ansarzadeh and colleagues, women’s age had an indirect effect on the quality of life through BMI, and a direct effect on the quality of life in GDM [43]. The relationship between obesity, GDM, and pregnancy outcomes can justify a low quality of life [78].

The finding indicated a significant negative correlation between the duration of marriage and GDMQoL scores. The negative effect of the duration of marriage on the quality of life was shown in some other studies [39, 79, 80]. According to a study, in the first 10 years of marriage, women experience a higher physical, mental, and environmental quality of life, and the duration of marriage can negatively affect various dimensions of quality of life [81]. Also, it seems increasing age can intensify the occurrence of adverse pregnancy complications and outcomes.

There was a significant positive association between the GDMQoL with the educational level of the women and also with the educational level of their spouses. Also, women’s education was a predictor of GDMQoL. A similar result was shown in some previous studies [46, 47, 82]. A study demonstrated that the level of education has a positive relationship with the mental health dimension of the quality of life in women with GDM [47]. Another study also demonstrated that pregnant women with higher education have higher scores for perceived mental health as well as perceived general health [83], while low education is a risk factor for impaired physical performance, which can affect the women’s quality of life [84].

We could not find a similar finding about the positive relationship between the spouse’s education and the quality-of-life score of women with GDM. It seems that the higher educational level of the spouse of women with GDM can be associated with a higher income level and more knowledge about care and support, which can have a positive effect on the women’s quality of life.

In our study, occupation was identified as an effective factor in the quality of life of women with GDM, so working women with GDM had a higher GDMQoL score than housewives. Also, occupation was a predictor for the GDMQoL score. Similarly, Kermansaravi and colleagues showed that working women scored higher than housewives in mental health and quality of life [85]. It is also demonstrated that education and occupation are the most important demographic factors in the physical dimension of the quality of life in pregnant women [86]. Educated and working mothers, due to their better socio-economic status, probably have a greater understanding of the importance of their health, and pay more attention to their appearance, weight control, and body mass index which can affect their health [87, 88].

There was a significant relationship between the husband’s occupation with the GDMQoL score. So the score of the GDMQoL of women whose husbands were employees was higher than the group whose husbands were workers. Some studies showed that the husband’s job is an effective factor in the quality of life of pregnant mothers [86, 89, 90]. Higher job levels may lead to positive economic consequences, increased social support, a greater variety of leisure activities, access to health services, and more academic-, social-, and family successes, which can increase happiness and Women’s mental health [91].

Also, our findings revealed that proper income and economic class of the family have a positive impact on GDMQoL. The result is in line with other studies [47, 77, 90, 92]. A higher quality of life was reported among women with GDM who had a high financial status. They had greater acceptance of illness which contributes to a higher quality of life and health status [59]. Although no study was found in women with GDM, the relationship between diabetes and the economic level of the family is numerous in the studies related to people with diabetes and they are indicative of the fact that the income level and economic conditions are among the variables related to the quality of life in diabetic women [77, 93–96]. High-income families can afford medical services without financial barriers [96] and so can directly support the quality of life of diabetic patients. However, low-income families are concerned about spending on medical expenses which can cause stress and negatively affect the psychological dimension of the quality of life. Also, income is associated with other social factors such as occupation, education, and health and so affects the quality of life [97]. Finally, sociodemographic factors such as occupation, education, social class, and income are closely related and can certainly affect the quality of life.

Finally, it can be suggested that future studies about improving the quality of life in women with GDM concentrate on the different interventions to promote mental health, such as comparative studies on the effectiveness of different treatment methods.

### Strengths and limitations

The use of GDMQoL-36 as a valid, reliable standard and a specific quality of life questionnaire for women with GDM instead of general questionnaires such as the World Health Organization quality of life questionnaire is the strength of this study.

A limitation of the study was that it was a cross-sectional study, which cannot dynamically describe the relationship between the quality of life and the duration of the disease in pregnant women with GDM. Also, the results of a cross-sectional study cannot judge precisely about cause-and-effect relationships of the variables.

Besides, the cross-sectional study makes it impossible to examine the longitudinal relationship between mental disorders such as depression, anxiety, and stress and GDMQoL. A path-analysis study by a social determinants approach and considering all associated factors are suggested for future studies.

## Conclusion

GDMQoL is correlated with mental disorders including depression, anxiety, and stress. Among them, depression is a predictor of the GDMQoL. Fetal-maternal health promotion is crucial for improving the quality of life of women with GDM, and so attention to the mental health of women with GDM should be considered as a priority. Healthcare providers can use cognitive behavioral therapy, mindfulness-based stress and anxiety reduction therapy, and other psychosocial counseling for women with GDM. These treatments can help pregnant women to reduce mental pressure and increase self-confidence in treatment. These interventions can prevent depression and thereby improve mental health and improve the quality of life during pregnancy.

This study showed that some personal and social characteristics including age, BMI, length of marriage, education, occupation, income, and economic class are associated with GDMQoL. Therefore, to improve the quality of life, possible measures such as optimal weight control, financial support, and providing free care in the future plans in GDM prenatal care services.

## Data Availability

The datasets used and/or analysed during the current study are available from the corresponding author on reasonable request.

## Abbreviations

GDM: Gestational diabetes mellitus
GDMQoL: GDM-related quality of life questionnaire
DASS: Depression, anxiety and stress scale
ADA: American diabetes association
LGA: Large for gestational age
BMI: Body mass index
DCQOL: Diabetes Clients Quality of Life questionnaire
S-CVI: Scale content validity index
S-CVR: Scale content validity ratio
ANOVA: Analysis of variance
SPSS − 23: Statistical Package for the Social Sciences (version 23)
